# Dermoscopy Training Course Improves Ophthalmologists’ Accuracy in Diagnosing Atypical Pigmented Periorbital Skin Lesions

**DOI:** 10.3390/diagnostics14222571

**Published:** 2024-11-15

**Authors:** Giovanni Rubegni, Alessandra Cartocci, Linda Tognetti, Matteo Orione, Caterina Gagliano, Tommaso Bacci, Antonio Tarantello, Nicola Lo Russo, Mario Fruschelli, Niccolò Castellino, Ernesto De Piano, Martina D’Onghia, Gabriele Cevenini, Teresio Avitabile, Pietro Rubegni, Alessio Luschi, Gian Marco Tosi

**Affiliations:** 1 Ophthalmology Unit, Department of Medicine, Surgery and Neuroscience, University of Siena, 53100 Siena, Italy; 2 Dermatology Unit, Department of Medicine, Surgery and Neuroscience, University of Siena, 53100 Siena, Italy; 3 Department of Ophthalmology, University of Catania, 95123 Catania, Italy; 4 Department of Medicine and Surgery, University of Enna “Kore”, 94100 Enna, Italy; 5 Department of Medical Biotechnologies, University of Siena, 53100 Siena, Italy

**Keywords:** eyelid skin lesions, dermoscopy, ophthalmology, lentigo maligna, pigmented periorbital lesions

## Abstract

Background/Objectives: Facial pigmented skin lesions are extremely common, starting from the fourth to fifth decades, especially in South-European countries, often located in the periorbital region. These include malignant forms, Lentigo maligna (LM) and lentigo maligna melanoma (LMM), characterized by growing incidence, and a series of benign simulators, including solar lentigo (SL), pigmented actinic keratosis (PAK), seborrheic keratosis (SK) and lichen planus-like keratosis (LPK). The clinical differential diagnosis of atypical pigmented skin lesions (aPFLs) can be difficult, even for dermatologists, leading to inappropriate skin biopsies with consequent aesthetic impacts. Dermoscopy of the facial area is a specific dermoscopic field that requires dedicated training and proved to increase diagnostic accuracy in dermatologists. Since these lesions are often seen by ophthalmologists at first, we aimed to evaluate the effect of a focused dermoscopy training course on a group of ophthalmologists naïve to the use of a dermatoscope. Methods: A set of 80 periorbital pigmented skin lesions with both clinical and dermoscopic images was selected and evaluated by six ophthalmologists before and after a one-day intensive dermoscopic training course. They were required to evaluate 80 periorbital lesions one month before and after a one-day intensive dermoscopic training course, illustrating second-level diagnostic options such as reflectance confocal microscopy (RCM), obtaining a total of 480 evaluations. Specifically, they had to provide, for each case, a punctual diagnosis and a management option among dermoscopic follow-up/skin biopsy/RCM/LC-OCT. Descriptive statistics were carried out, and the accuracy (ACC), sensitivity (SE), and specificity (SP), with their 95% confidence interval (95% CI), were estimated. Results: In the pre-course test, ophthalmologists achieved 84.0% SP, 33.3% SE and 63.7% ACC, while after the course, SE increased by +9% (i.e., 41.7%), SP decreased by 4%, and ACC remained comparable, i.e., 64.6%. In the management study, the percentage of benign lesions for which a close dermoscopic follow-up was suggested significantly decreased (51.6% versus 22.2%), in parallel with an increase in the number of lesions referred for RCM. As for malignant cases, the reduction in responses “close dermoscopic follow-up” decreased from 37.0% to 9.9%, (−27%), in favor of RCM (+15%) and skin biopsy (+12%). Conclusions: The ophthalmologists proved to be very receptive in quickly metabolizing and putting into practice the concepts learned during the one-day intensive dermoscopy training course. Indeed, after only a one-day lesson, they were able to increase their SE by 9% and to improve their management strategy. The present findings highlight the importance of providing training ophthalmologists in dermoscopy during residency programs, in terms of benefits for the correct patient care.

## 1. Introduction

According to the literature, 5–10% of all skin cancers are located in the periorbital region [1,2,3,4,5,6]. Ophthalmologists are healthcare professionals involved in the prevention, diagnosis, and treatment of diseases and injuries of the eye and associated structures. They represent, in many cases, the first professional evaluating the periorbital region and routinely examine a larger number of patients with these kinds of lesions, compared to other healthcare professionals. As such, ophthalmologists are a targetable group for the early detection of periorbital melanoma, therefore potentially improving overall and specific survival outcomes in these patients.

However, there is a series of benign simulators, including solar lentigo (SL), pigmented actinic keratosis (PAK), seborrheic keratosis (SK), and lichen planus-like keratosis (LPK), that can clinically simulate malignant melanocytic facial lesions (i.e., LM, LMM). The clinical differential diagnosis between these benign and malignant atypical pigmented skin lesions (aPFLs) can be difficult, even for dermatologists, leading to inappropriate skin biopsies with a consequent aesthetic impact.

Dermoscopy is well established among dermatologists as an assessment tool and is a non-invasive technique for the visual inspection of skin lesions. A dermatoscope is a hand-held device that contains an integral light source and typically a 10–20× magnification lens [7]. A dermatoscope acts to magnify a lesion and reduce the interference of light reflection from the skin’s surface, to allow pigmented and vascular structures in the deep epidermis and superficial dermis to be visualized [8,9]. There is evidence from systematic reviews that skin lesion assessment using dermoscopy is more accurate than visual inspection alone for the detection of melanomas [10,11]. However, there is also evidence that this improvement in diagnostic accuracy is only observed when used by doctors who are experienced in its use, highlighting the necessity of dermoscopy training [12,13]. Evidence from the literature would suggest that the use of dermoscopy among physicians could improve their triage and assessment of skin lesions, reducing unnecessary excisions or referrals [14,15,16]. Despite this, dermoscopy is used by a small minority of non-dermatologists in many countries including the UK, USA, and France [17]. Finally, dermoscopy of the facial area is a specific dermoscopic filed that requires dedicated training and is proved to increase diagnostic accuracy in dermatologists

In the last few decades, the non-invasive differential diagnosis of aPFLs has benefited from the introduction of a new imaging technology capable of achieving cellular resolution, namely reflectance confocal microscopy (RCM), that provides in vivo horizontal histology-like images and significantly improves LM detection [18].

The aim of this study was to examine the effects on periorbital skin lesion diagnostic accuracy of a 1-day “full-immersion” training course in dermoscopic RCM imaging among Italian residents and specialists in ophthalmology.

## 2. Materials and Methods

### 2.1. Online Testing

Six ophthalmologists with no experience in dermoscopy were recruited for this study (GR, MO, NC, NL, AT, CG); in particular, two of them were residents with less than four years of experience in ophthalmology. The six ophthalmologists participated in a dedicated and specific 1-day course on periorbital lesions carried out by a dermatologist with long-lasting experience in face dermoscopy (LT).

The six ophthalmologists were required to evaluate 80 periorbital lesions, both one month before and one month after the course, obtaining a total of 480 evaluations. Specifically, they had to provide a diagnosis of benign or malignant lesion and determine the appropriate management of each lesion among close “*dermoscopic follow-up*”, “*perfoming an RCM*” or “*taking a skin biopsy*” (for histopathological examination).

### 2.2. Dermoscopic Training Course

The course comprised 5 separate lectures:

- New techniques for non-invasive diagnosis in dermatology (RCM and line-field confocal optical coherence tomography) (60 min);
- Dermoscopic technique and terminology (60 min);
- Introduction to “pattern analysis” and the diagnosis of periorbital benign non-melanocytic lesions (60 min);
- Dermoscopic diagnosis of melanocytic lesions (180 min) (these lectures were interlaced with 2 case-based interactive sessions (45 and 60 min, respectively)).

Then, the course was concluded with a question-and-answer session (80 min). All participants were provided with PDF files of the presentations.

### 2.3. Case Study

**Figure 1:**
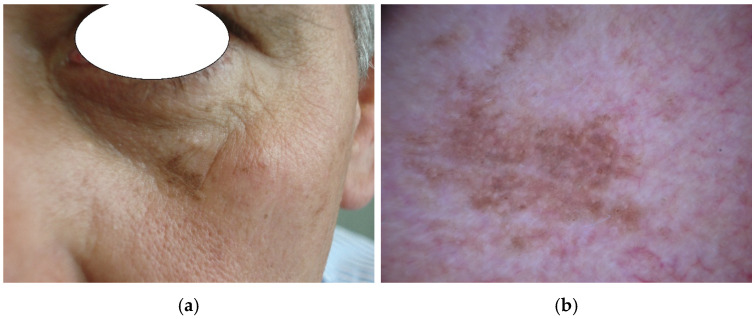

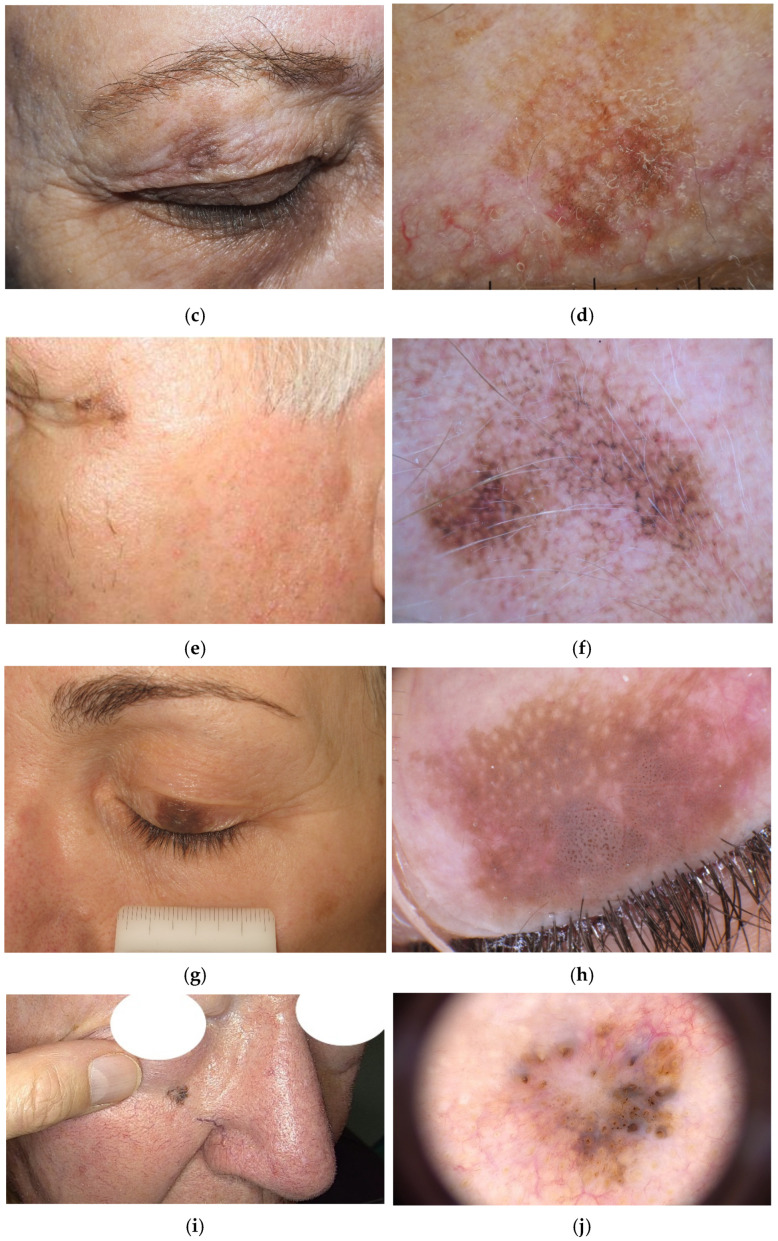
Example of different atypical pigmented facial lesions (aPFL) of the periorbital region with similar clinical appearance: macroscopic clinical images (**a**,**c**,**e**,**g**,**i**) and corresponding dermoscopic images (**b**,**d**,**f**,**h**,**j**) taken with polarized dermoscopy, OM 20X. Three aPFL lesions appearing as brownish macule and similar dimensions: a pigmented actinic keratosis in a 50-year-old male, with 12 mm maximum diameter (**a**), showing an homogenous reticular pattern sparing the follicular openings (**b**); a solar lentigo in a 71-year-old woman, with 7 mm maximum diameter, showing yellowish-brownish homogenous pigmentation around the hair follicoles (**d**); a lentigo maligna in a 66-year-old male, with 9 mm maximum diameter, showing granular brownish-grey pattern and polygonal structures involving follicular openings (**f**). A 12 mm seborrheic keratosis combined with a solar lentigo in a 59-year-old woman (**g**) showing cerebriform pattern and comedo-like openings in the inferior part (**h**). An 8 mm lentigo maligna melanoma in an 87-year-old male (**i**) showing asymmetry of structures and colors, granular pattern, blue-grey areas and evident involvement of the follicular openings (**j**).

The 80 lesions of the periorbital area (Figure 1) were derived from the *iDScore-facial database*, as previously described [18,19,20]. Briefly, an international clinico-dermoscopic web registry was used, including 1197 challenging aPFLs created by dermatologists of the European Teledermatology Task Force (EADV) (LT, PR) and bioengineers of Siena University (AC, AT, GC) in collaboration with 11 Dermatologic Centres dedicated to skin cancer screening [21].

Each aPFL was composed of one dermoscopic image, one clinical image, and several patients’ and lesion information (i.e., maximum diameter of the lesion, patient sex (F/M) and age (years); for LMM: thickness, mythoses number, regression %, presence of lymphocytic infiltrate).

Thus, 32 out of 80 lesions (40.0%) were LM/LMM, and 48 (60%) were benign (8 (10.0%) pigmented actinic keratosis, 7 (8.8%) atypical nevi, 26 (32.5%) were solar lentigo, 3 (3.8%) were seborrheic keratosis, and 4 (5.0%) were seborrheic lichenoid keratosis). The histological diagnosis was blinded until the end of the post-course evaluation.

### 2.4. Ethics

This study was approved by Siena local ethical committee in April 2018 (Azienda Ospedaliero-Universitaria Senese, Siena, Italy, Study Protocol No. 16801) and then shared with the participating centers, following the recommendations from the Declaration of Helsinki. All data were deidentified before use and kept in accordance with the EU General Data Protection Regulations (GDPR) on the processing of personal data and the protection of privacy in electronic communications (2016/679/EU) [21].

### 2.5. Statistical Analysis

Descriptive statistics were carried out; absolute frequencies and percentages were estimated for qualitative variables and mean and standard deviation for the quantitative ones. T student test and chi squared test were performed to compare LM cases with benign cases. To compare pre- with post-course management, Bowker test and post hoc analysis based on multiple McNemar test with false-discovery rate correction were performed. Sensitivity, specificity, and accuracy were estimated and compared between pre- and post-course by proportion test. *p* < 0.05 was considered statistically significant. All the analyses were carried out with R version 4.3.1.

## 3. Results

### 3.1. Study Population

This study included a total of 80 patients, with 48 (60%) benign lesions and 32 (40%) lentigo maligna/lentigo maligna melanoma (see Table 1). The mean age of patients was significantly different (*p* = 0.010) between the two groups, with those in the LM group being older on average (66.53 ± 12.24 years) compared to the benign group (59.21 ± 11.89 years). The proportion of male patients was not significantly different between the groups, with 29.8% in the benign group and 37.5% in the LM group (*p* = 0.637). The mean lesion diameter was similar between the groups, with 10.68 ± 6.50 mm in the BEN group and 11.00 ± 4.53 mm in the LM group, showing no statistically significant difference (*p* = 0.810) (see Table 1).

### 3.2. Diagnostic Accuracy of Periorbital Skin Lesions Pre- and Post-Course

In the pre-course test, ophthalmologists achieved a specificity of 84.0%, a sensitivity of 33.3%, and an accuracy of 63.7%. Indeed, they correctly diagnosed 64 cases of LM, while they misdiagnosed 46 cases; conversely, they correctly diagnosed 241 cases as benign, while they classified a total of 128 LM cases as “benign”.

One month after the course, the sensitivity increased, rising by 9% (from 33.3% to 41.7%), even though it was not significant (*p* = 0.114). The specificity decreased slightly, though not significantly (*p* = 0.280), by about 4%. Indeed, participants correctly diagnosed 80 cases of LM, while they misdiagnosed 58 LM cases. On the other hand, they correctly diagnosed 230 cases as benign, while they classified as “benign” a total of 112 LM cases.

**Figure 2:**
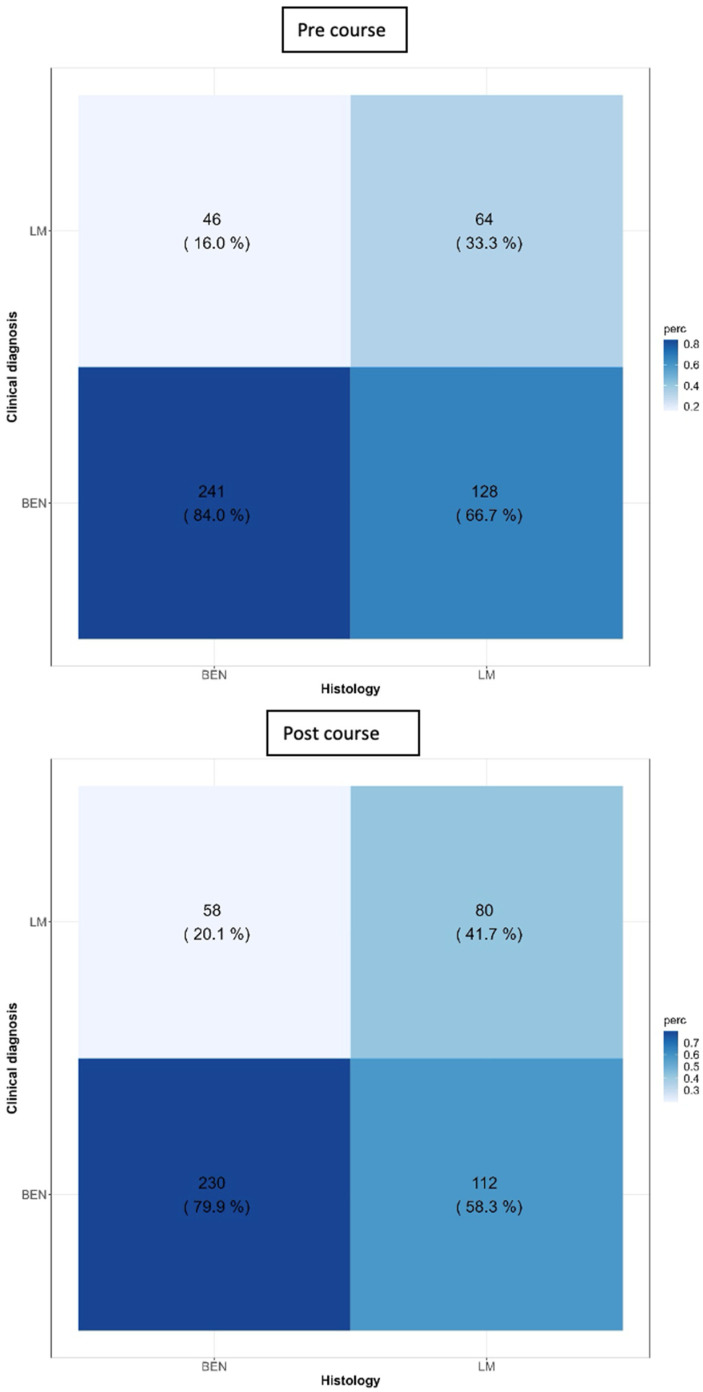
Accuracy of ophthalmologists’ diagnoses before and after the course.

Overall, the accuracy remained comparable at 64.6% (*p* = 0.788) (Figure 2).

### 3.3. Management of Periorbital Skin Lesions Pre- and Post-Course

**Figure 3:**
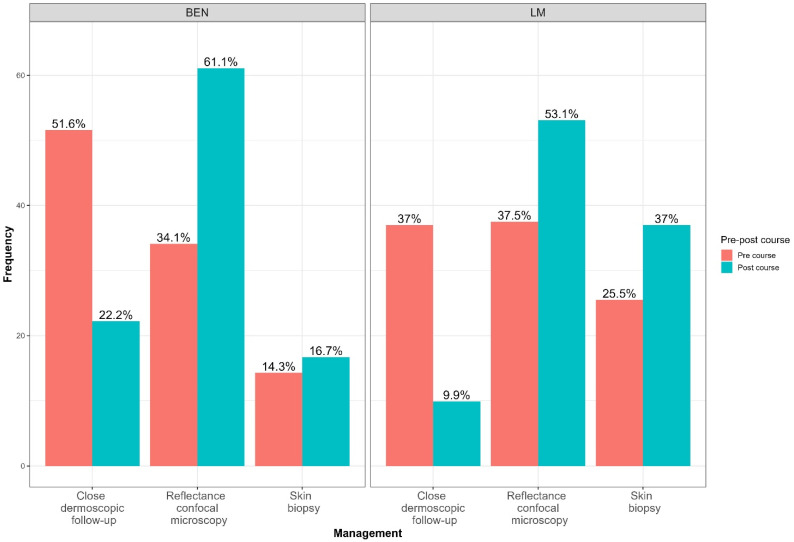
Ophthalmologists suggested management of periorbital pigmented skin lesions before and after the course.

The ophthalmologist’s management suggestions are shown in Figure 3, comparing the pre-course and post-course data. It can be observed that for benign cases, the percentage of lesions for which a *close dermoscopic follow-up* was suggested significantly decreased from 51.6% to 22.2% (*p* < 0.001). This reduction of about 30% is accompanied by a significant increase in the number of lesions referred for further non-invasive diagnostic examination (*p* < 0.001). As for LM, the reduction in images requiring “close dermoscopic follow-up” decreased significantly from 37.0% to 9.9% (*p* < 0.001), representing a 27% reduction. The lesions referred for RCM increased by 15%, but, more importantly, there was a 12% increase in correctly excised LM.

## 4. Discussion

Lentigo maligna (LM) is an in situ cutaneous melanoma occurring in photodamaged skin, with a peak incidence in the 7–8th decades of life, characterized by the proliferation of atypical melanocytes along the basal epidermal layer. If untreated, LM progresses to its invasive form, lentigo maligna melanoma (LMM), in about 2–3.5% cases per year [1]. The great majority of LM/LMM cases localized on the face, scalp, and neck, with rare presentations on the trunk and extremities [2]. Compared to other subtypes of cutaneous melanoma, melanomas of the head and neck area are much more challenging to diagnose and have a worse prognosis, with significantly lower 5-year melanoma-specific survival rates [3,4]. This diagnostic difficulty is due to the fact that photoaged skin also exhibits a series of benign lesions that can possibly simulate LM [5].

Nowadays, the differential diagnosis of LM/LMM from benign pigmented simulators of the periorbital area presents a challenge in clinical practice for dermatologists and ophthalmologists [22,23]. Given that a diagnosis of malignancy will require demolitive surgery, for this reason, a timely and correct recognition when the lesion is limited in dimension would be desirable [22,23,24]. The increasing use of portable dermatoscopes largely helped in this sense [25,26,27], especially when dealing with “difficult” lesions that appear equivocal due to unspecific dermoscopic patterns or very small dimensions [20,28,29]. In this sense, the introduction of new techniques able to investigate lesions of a few millimeters reaching cellular resolution, like RCM, significantly impacted this early detection, currently regarded as a second-line diagnostic step [30,31]. However, both these techniques require knowledge from the physician who first screens the patient and need to be performed by experts in the field. On the other hand, we have to consider that a proper dermoscopic screening of a pigmented facial lesion can reduce the number needed to biopsy, that is, the number of false-positive biopsies performed, the inverse of positive predictive value, to find a melanoma. This performance indicator is particularly important in the field of facial lesions given the progressive access for physician evaluation due to global population ageing. For melanoma in general, studies from the United States report ratios between 6.91 and 7.5 needed for biopsy (NBB) [32,33]. For facial lesions, these NNB values turned out to be higher, even for expert dermatologists, according to the findings of tele-dermoscopic studies performed on the *iDScore facial dataset* [28]. Finally, even the histology technique, which reset the gold standard of dermatologic diagnosis, itself has some limitations, especially in the differentiation between an early LM and UV-induced melanocytic hyperplasia in chronically sun damaged skin [31].

This is the first study reporting the before-and-after performance of a pool of variously experienced ophthalmologists, properly trained and tested over a selected pool of aPFLs of the whole face, considered “difficult” even for dermatologists (i.e., average accuracy 43%) [28].

Based on the present findings, ophthalmologists proved to be able to quickly metabolize and put into practice the concepts learned during the one-day intensive dermoscopy training course. Indeed, after only a one-day lesson, they were able to increase their sensitivity by 9% and to improve their management strategy. Actually, in the pre-course test, the ophthalmologists achieved 84.0% specificity, 33.3% sensitivity, and 63.7% accuracy, while after the course, sensitivity increased by +9% (i.e., 41.7%), specificity decreased by 4%, and accuracy remained comparable, i.e., 64.6%. The increase in sensitivity basically derives from the knowledge acquisition in dermoscopic pattern analysis of malignant forms, while the decrease in specificity reflects the knowledge implementation in the spectrum of benign cases of atypical appearance.

Another factor that should be considered is that the diagnosis in a tele-dermatologic online setting, differing in many respects from in vivo diagnosis, where a full-patient examination is possible, and this may affect the final accuracy value.

The training on the first- and second-level diagnostic techniques, finally, produced an increase in the number of appropriate management responses versus inappropriate ones. Indeed, in the management study, the percentage of benign lesions for which a close dermoscopic follow-up was suggested significantly decreased (51.6% versus 22.2%), in parallel with an increase in the number of lesions referred for RCM. As for malignant cases, the response “close dermoscopic follow-up” decreased from 37.0% to 9.9%, (−27%), in favor of RCM (+15%) and skin biopsy (+12%).

Finally, it is worth noting that the diagnostic performances obtained by ophthalmologists were not significantly inferior to that of dermatologists [20]. Indeed, we previously compared the responses given after the course by the six opthalmologists with that of two dermatologists of different experience levels (one specialist and one dermatology resident) on the same pool of 79 orbital/periorbital lesions from the *iDScore-facial database*; all responses were obtained by using the same online testing modality [20]. In this cross-analysis, the ophthalmologists’ accuracy was 63.50% (95% CI: 58.99–67.85%) versus 66.50% (95% CI: 58.5–73.8) of dermatologists and, thus, not significantly different. The sensitivity gap was significant, namely −13.3% (i.e., 33.3% of ophthalmologists versus 46.9% of dermatologists). Of note, the specificity of opthalmologists was slightly superior (84.0% (95% CI: 79.2–88.1%) compared to that of dermatologists, 79.7% (95% CI: 70.2–87.4%). Concerning the responses on lesion management, it appeared that dermatologists preferred a more “operative” experienced-based approach resulting in a request for biopsy in 24% benign cases and 50% malignant cases, while ophthalmologists preferred a more “conservative” approach (i.e., request for biopsy in 14.2% of benign cases and 25.5% of malignant cases), in favor of follow-up and RCM management options.

## 5. Conclusions

Providing ophthalmologists with specific dermoscopic/imaging training would be desirable to both catch those shares of LM/LMM that are evaluated by the eye specialist (instead of the dermatologists) and to reduce the inappropriate quote of skin biopsy for benign clinical simulators [28].

The present findings also highlight the “potentialities” of this medical figure in the management of facial lesions seen during screening for ophthalmologic issues, and they stress the importance of providing training for ophthalmologists in dermoscopy during residency programs, in terms of benefits for the correct patient care.

These results confirmed that dermoscopy training considerably improves the diagnostic accuracy and management of pigmented lesions. Encouraging universities to propose that general practician residents undergo training in dermoscopy could significantly improve skin cancer screening and, therefore, improve skin cancer survival.

In the near future, multiple data analyses based not only on the biological and anamnestic data of lesions and patients but also on the collected participants’ evaluations will allow for the development of different precious tools (i.e., semi-automatic scoring checklist, automatic AI softwares [34]) able to support physicians, more or less skilled in dermoscopy, in the diagnosis and management of aPFLs.

## Figures and Tables

**Table 1:** Distribution of patient and lesion characteristics.

	Total *n* = 80	BEN *n* = 48	LM *n* = 32	*p*-Value
Patient Age	62.18 ± 12.49	59.21 ± 11.89	66.53 ± 12.24	0.010
Patient Sex (Male)	26 (32.5%)	14 (29.8%)	12 (37.5%)	0.637
Lesion Maximum Diameter (mean (SD))	10.81 ± 5.75	10.68 ± 6.50	11.00 ± 4.53	0.810

## Data Availability

Data available upon request.
